# Are the Post-transplant Outcomes of Kasai's Early Failure and Late Failure Comparable to the Primary Liver Transplantation?

**DOI:** 10.7759/cureus.51424

**Published:** 2023-12-31

**Authors:** Valberto Sanha, Tales A Franzini, Waldemir F Junior, Antonio N Kalil

**Affiliations:** 1 General Surgery, Federal University of Health Science of Porto Alegre, Porto Alegre, BRA; 2 Medicine, Federal University of Health Science of Porto Alegre, Porto Alegre, BRA; 3 Surgery, Federal University of Health Science of Porto Alegre, Porto Alegre, BRA; 4 General Surgery and Hepato-Pancreato-Biliary (HPB) & Transplant Surgery, Federal University of Health Science of Porto Alegre, Porto Alegre, BRA

**Keywords:** biliary atresia, kasai hepatico-portoenterostomy, kasai procedure, pediatric liver transplant, primary liver transplantation

## Abstract

It is uncertain whether prior Kasai procedures negatively impact the outcomes of liver transplantation (LT). The prior meta-analysis did not distinguish between Kasai early failure (K-EF) and late failure (K-LF). Numerous studies have been recently published; therefore, we perform a systematic review and meta-analysis.

We searched PubMed and Embase databases to identify studies comparing the outcomes of biliary atresia (BA) patients undergoing primary LT versus patients with prior Kasai procedures. Subgroup analysis was done at the time of Kasai failure (early vs. late).

Twenty-five studies comprising 6,408 patients receiving LT were included in the analysis. We found a statistically significant increase in one-year graft survival in K-LF versus primary liver transplant (pLT) (P = 0.0003). One-year patient survival was also increased in K-LF, although not statistically significant (P = 0.09). No difference in the one- and five-year graft and patient survival, reoperation rate, infection, and biliary complication was seen in pLT vs overall prior Kasai (K-EF and K-LF).

These results suggest that prior kasai procedure does not negatively impact the outcome of LT. In addition, BA patients with prior Kasai undergoing LT later in life tend to perform better than primary liver transplants.

## Introduction and background

Biliary atresia (BA) is the major cause of pediatric end-stage liver disease and a common indication for liver transplants worldwide. It is characterized by obliteration of bile ducts; the exact cause of BA is not well established. BA has a worldwide distribution, affecting different ethnicities. The Kasai procedure remains the initial and standard treatment for infants with BA. However, the procedure has a high failure rate. There is concern about increased surgical complexity during salvage LT for patients with prior portoenterostomy, subsequently impacting the postsurgical outcomes in this population [1,2]. Moreover, over half of BA infants with prior Kasai procedures will eventually be listed for LT [3], and the outcomes of liver transplants have dramatically improved in recent eras, which led to the hypothesis of primary liver transplant instead of initial Kasai portoenterostomy in selected infants with BA [2,4].

It is debatable whether prior portoenterostomy has a negative impact on the outcome of LT. Previous studies have reported conflicting outcomes [1,2,4-7]. A prior meta-analysis of 1,560 patients reported comparable patient and graft survival between patients with primary LT (pLT) and prior portoenterostomy, and reduced infection rate in the pLT group [8]. However, there was no distinction between patients with prior Kasai procedures who had early portoenterostomy failure (failure within one year of the procedure) and late Kasai failure (after the first year of the procedure). Studies have shown differences in the clinical characteristics and outcomes between these two distinct groups [1,5-7].

The impact of prior portoenterostomy on LT for BA remains controversial. Several recent studies on this theme have been published. Therefore, we performed an updated systematic review and meta-analysis and explored the subgroup analysis of patients who had Kasai early failure (K-EF), Kasai late failure (K-LF), and pLT.

## Review

Methodology

Search Strategy

This systematic review and meta-analysis were conducted according to the recommendations of the Cochrane Collaboration and the Preferred Reporting Items for Systematic Reviews and Meta-Analysis (PRISMA) statement guidelines [9]. Two authors (V.S. and W.J.) independently reviewed the literature. Disagreements were resolved among three authors (V.S., W.J., and T.F.). The pre-specified research protocol was not published. A systematic search was performed in PubMed and EMBASE databases from inception to April 2023 for studies published with the following heading terms: “Biliary atresia,” ‘transplantation,” “liver transplantation,” “hepatic transplantation,” “portoenterostomy,” “Kasai hepatoportoenterostomy,” “Kasai procedure,” and “biliary-enteric drainage.” In addition, the references of the retrieved studies were evaluated for additional studies. There were no restrictions on the language and publication year.

Selection Criteria

Studies that met the following criteria were included (1) studies with BA patients; (2) those comparing the outcome of LT in BA patients with prior Kasai procedure versus BA patients without prior Kasai procedure; and (3) reported at least one of the outcomes of interest. Reviews, case reports, and editorials were excluded.

Endpoints

The authors independently extracted baseline characteristics and the endpoints included for the analysis using pre-specified criteria were search, data extraction, and quality assessment. We performed a systematic review and meta-analysis on the following endpoints: one-year and five-year patient survival, one-year and five-year graft survival rate, biliary complication, vascular complication, infection, reoperation rate, retransplantation, and length of hospital stay. We compared these outcomes between patients who had prior Kasai vs. pLT. We further subdivided the prior Kasai group into two groups for subgroup analysis. K-EF when a liver transplant was performed within one year of the initial Kasai procedure, and K-LF when LT was performed beyond the first year of the initial Kasai. Each of these groups was compared with the pLT group.

*Quality Assessment* 

The risk of bias and quality assessment of each individual study was performed independently. Quality assessments of non-randomized controlled trials were appraised using the Cochrane Collaboration tool for assessing the risk of bias in non-randomized studies (ROBINS-I) [10]. Each study was categorized as critical, serious, moderate, and low risk in all seven domains: confounding, selection, classification, deviations from intended interventions, missing data, measurement of the outcomes, and selection of reported results. Publication bias was investigated with funnel-plot analysis of the primary outcomes.

Statistical Analysis

This systematic review and meta-analysis was performed according to the recommendations of the Cochrane Collaboration and PRISMA statement guidelines. For binary outcomes, the effect of the intervention was compared using pooled odds ratios (OR) with 95% confidence intervals (CI), and for continuous outcomes, weighted mean differences were used. Heterogeneity was evaluated with the Cochran Q test and I² statistics; p-values < 0.10 and I² > 25% were considered significant for heterogeneity. Random-effects models were used in pooled outcomes with high heterogeneity, and a fixed-effect model was used for endpoints with I² < 25%, low heterogeneity. We used Review Manager 5.4 (Nordic Cochrane Centre, The Cochrane Collaboration, Copenhagen, Denmark) for statistical analysis.

Results

Study Selection and Characteristics

**Figure 1:**
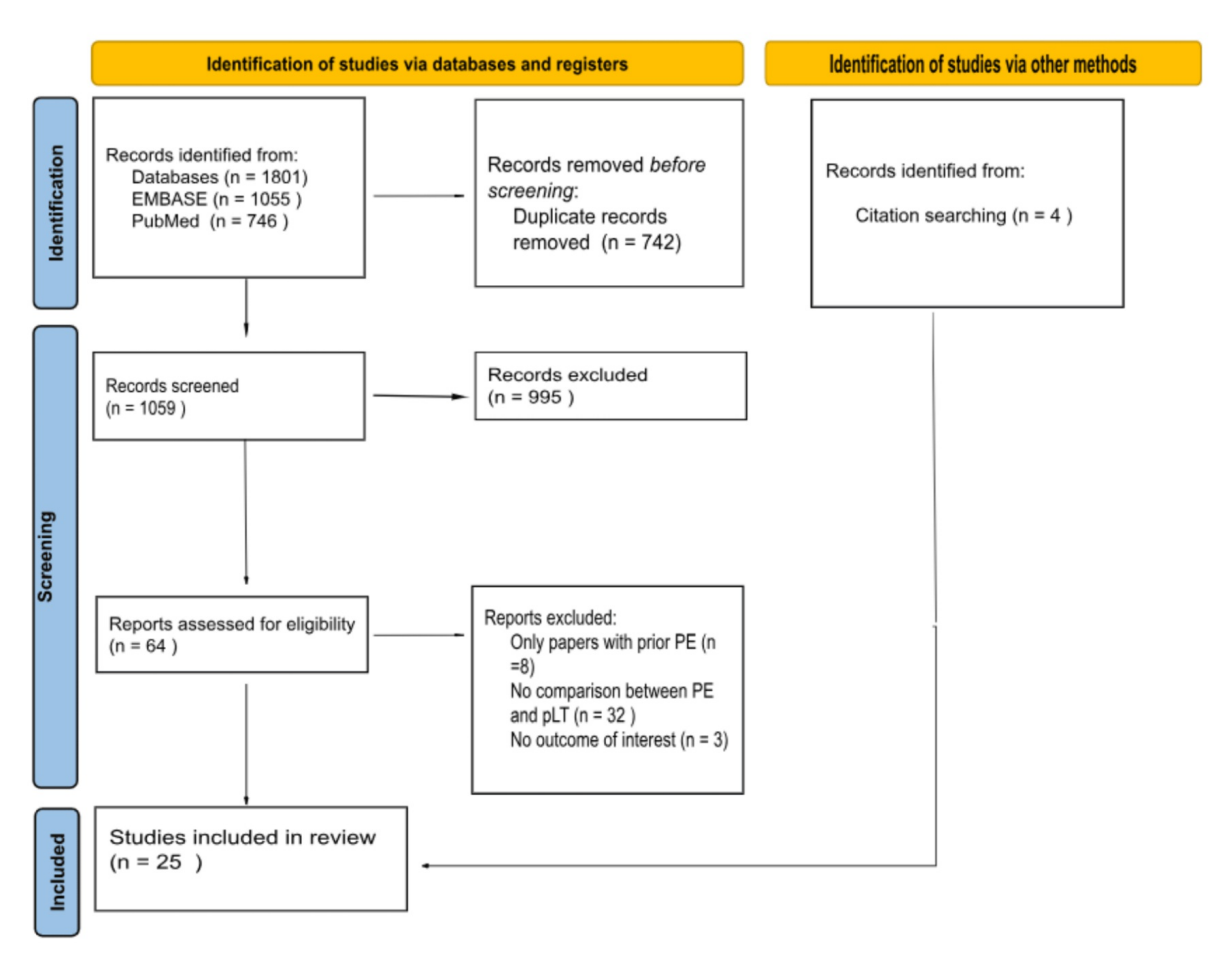
PRISMA flow diagram

The initial search yielded 1,801 results; 64 studies remained for full-text review after duplicate removal and exclusion of studies that did not meet the inclusion criteria based on title and abstract screening. We finally included 25 studies [1-7,11-28], of whom four were identified from relevant references and met the inclusion criteria (Figure 1). All studies were non-randomized, comprising 6,408 BA patients, 22% were primary liver transplants and 72% had prior portoenterostomy. Table 1 summarizes the pretransplantation baseline characteristics between pLT and the prior Kasai group.

**Table 1:** Baseline characteristics of included studies PE - portoenterostomy, Non-PE - non-portoenterostomy, LT - liver transplant, LD - living donor, DD - deceased donor

	Number of patients (%)			Age at LT (days) N (variation range)			LT type (LD:DD)			PELD score - mean (SD or range)			LT operation Time (min) - (SD or range)			Intraoperative blood loss (mL) - (variation range)			Overall survival % (y for each study)			ICU stay (days) - (SD or range)			Post-op MV (days)			Vascular Complication			Biliary complication			Hospital stay (days) - (SD or range)			Rejection			Infection Rate		
Study	PE	Non-PE	Total	PE	Non-PE	p-value	KP	Non-KP	Total	PE	Non-PE	p-value	PE	Non-PE	p-value	PE	Non-PE	p-value	PE	Non-PE	p-value	PE	Non-PE	p-value	PE	Non-PE	Total	PE	Non-PE	Total	PE	Non-PE	Total	PE	Non-PE	p-value	PE	Non-PE	p-value	PE	Non-PE	p-value
Alexopoulos et al., 2012 [1]	112 (83.58%)	22 (16.42%)	134	609±933	333 ±149	-	-	-	-	-	13.6 ± 6.0	-	396 ± 108	408 ± 120	-	-	-	-	87.5% (1y)	100% (1y)	0.05	-	-	-	-	-	-	-	-	-	3/112	3/22	-	-	-	-	-	-	-	53/112	6/22	-
LeeVan et al., 2018 [2]	147 (31.95%)	313 (68.05%)	460	313 (221-505)	315 (230-485)	0.8	-	-	-	-	-	-	-	-	-	-	-	-	94% (1y); 95% (5y); 88% (10y); 94% (15y)	87% (1y); 91% (5y); 85% (10y); 92% (15y)	0.01	-	-	-	-	-	-	-	-	-	-	-	-	-	-	-	-	-	-	-	-	-
Sandler et al., 1996 [4]	49 (85.9%)	8 (14.1%)	57	839.5±146	255.5±73	0.06	(00:49)	(00:8)	(00:57)	-	-	-	593.9± 29.3	476.8± 53.3	0.06	-	-	-	74.4% (1y)	75% (1y)	0.8	-	-	-	-	-	-	7	0	7	3/49	1/8	4	-	-	-	19(38.78%)	3(37.5%)	0.9	-	-	-
Yoeli et al., 2022 [5]	2862 (84.54%)	523 (15.45%)	3385	58.4	84	<0.001	(380:1498)	(69:366)	(449:1864)	17.83	19.01	<0.001	-	-	-	-	-	-	96% (1y);95% (3y); 95% (5y)	96% (1y); 93% (3y); 93% (5y)	0.2	-	-	-	-	-	-	-	-	-	-	-	-	24.22d	25.48d	<0.001	-	-	-	-	-	-
Neto et al., 2015 [6]	209 (60%)	138 (40%)	347	690±849	324±150	<0.001	(189:12)	(120:10)	(309:22)	15.5±8.6	19.2±7.6	<0.001	-	-	-	-	-	-	-	-	-	2.5 (1-14)	4 (2 to 7)	0.06	-	-	-	90	32	122	39/209	12/138	51	10 (6-16)	11 (7 to 17)	0.74	-	-	-	-	-	-
Liu et al., 2022 [7]	154 (78.2%): 71 <1y KP-EF; 59 1~5y KP-MF; 24 >5y KP-LF	43 (21.8%)	197	231 <1y KP-EF; 687d 1~5y KP-MF; 2466 >5y KP-LF	192 (159-240)	<0.001	59:12 for <1y KP-EF; 45:14 for 1~5y KP-MF; 13:11 for >5y KP-LF	(37:6)	(154:43)	17 (12-25) for <1y KP-EF; 1 (−2 to 5) for 1~5y KP-MF; 1 (−1 to 4) for >5y KP-LF	20 (15-27)	<0.001	375 (350-435) for <1y KP-EF; 380 (325-435) for 1~5y KP-MF; 398 (327-480) for >5y KP-LF	390 (350-440)	0.66	150 (100-300) for <1y; 120 (100-200) for 1~5y; 200 (124-338) for >5y	150mL (100-250)	0.07	97.4% (5y)	93% (5y)	0.02	3.8 (2.9-4.9) for <1y KP-EF; 3.6 (2.6-4.6) for 1~5y KP-MF; 3.1 (1.9-3.8) for >5y KP-LF	3.9 (2.3-5.6)	0.01	-	-	-	22	8	30	15/154	6/43		32 (24-47) (for <1y) KP-EF; 23d (18-32) (for 1~5y) KP-MF; 23d (15-35) (for >5y) KP-LF	30d (21-47)	<0.001	1.4% in KP-EF; 1.7% in KP-MF; 12.5% in KP-LF	0%	0.02	-	-	-
Millis et al., 1998 [11]	28 (78%)	8 (22%)	36	960	600	<0.05	(0:28)	(0:8)	(0:36)	-	-	-	-	-	-	5.6 (0.44–33) Vol	4.1 (1.2–13) Blood volumes		82% (6mo)	63% (6mo)		-	-	-	-	-	-	-	-		6/28	1/8	(7/36)	-	-	-	-	-	-	10/28	2/8	-
Wood et al., 1990 [12]	46 (95.8%)	2 (4.2%)	48	-	-	-	-	-	-	-	-	-	-	-	-	-	-	-	-	-	-	-	-	-	-	-	-	-	-	-	-	-	-	-	-	-	-	-	-	-	-	-
Meister et al., 1993 [13]	32 (82%)	7 (18%)	39	495	180	0.013	(0:32)	(0:7)	(0:39)	-	-	-	641.4	552.6	0.025	-	-	-	90% (1y)	100% (1y)		-	-	-	-	-	-	-	-	-	2/32	1/7	3/39	31.7	44	0.18	-	-	-	9/32	1/7	-
Chardot et al., 2001 [14]	208 (93%)	17 (7%)	225	-	-	-	-	-	-	-	-	-	-	-	-	-	-	-	70.1% (5y)	76.4% (5y)	-	-	-	-	-	-	-	-	-	-	-	-	-	-	-	-	-	-	-	-	-	-
Diem et al., 2003 [15]	285 (86.98%)	43 (13.12%)	328	-	-	-	-	-	(59:269)	-	-	-	-	-	-	-	-	-	87.2 %(1y); 82.5%(5y)	90.3% (1y); 86.4% (5y)	-	-	-	-	-	-	-	-	-	-	-	-	-	-	-	-	-	-	-	-	-	-
Visser et al., 2004 [16]	53 (80.3%)	13 (19.7%)	66	870±1470	360±240		(24:29)	(7:6)	(31:35)	9.7 ± 9.2	13.7 ± 6.4	0.075	395 ± 106	400 ± 110		1400 ± 2900 mL	500 ± 200 mL	0.086	-	-	-	-	-	-	-	-	-	-	-	18	-	-	-	-	-	-	-	-	-	-	-	-
Cowles et al., 2008 [17]	61 (86%)	10 (14%)	71	258	237	-	-	-	32::39	14	15.5	-	-	-	-	-	-	-	93.4%(5y)	100% (5y)	-	-	-	-	-	-	-	-	-	-	-	-	-	-	-	-	-	-	-	-	-	-
Tiao et al., 2008 [18]	60 (56.6%)	46 (43.4%)	106	2007.5±2299.5	(584±401.5)	< .001	(00:60)	(00:46)	(00:106)	-	-	-	-	-	-	-	-	-	94.9% (5y)	97.8% (5y)	0.16	-	-	-	-	-	-	-	-	-	-	-	-	-	-	-	-	-	-	-	-	-
Sui et al., 2014 [19]	28 (53%)	25 (47%)	53	375	225	-	28:0	25:0	52:0	12.3 ± 10.9	22.1 ± 19	-	521.5 ± 65.6	573.2 ± 98.5		351.8 ± 208.8 mL	277.6 ± 133.9 mL	-	-	-	-	-	-	-	-	-	-	-	-	-	-	-	-	-	-	-	-	-	-	12/28	10/25	-
Li et al., 2019 [20]	89 (59.33%)	61 (40.66%)	150	471 ±483	252±156	0.000⁎	(89:00)	(61:00)	(150:00)	15.51 ± 7.92	18.44 ± 6.97	0.409	552 ± 102	540 ± 102	0.812	335.6 ± 219.3 mL total	321.1 ± 193.3	0.475	95.4% (1y)	96.7%(1y)		3.5d(3–5)	3.7(3.3–4.8)	0.084	4.1h(2–7.1)	5.2h(2.9–10.8)	0.173	5/89	6/61	11/150	9/89	5/61	14/150	28d(22.3–44.8)	29(19–53)	0.937	-	-	-	-	-	-
Tambucci et al., 2021 [21]	296 (75.3%)	97 (24.7%)	393	416 (277–759)	310.25 (237.25–474.5)	<0.0001	(185:111)	(87:10)	(272:121)	12.9 (7.1–20.8)	18.0 (13.5–23.6)	<0.0001	-	-	-	-	-	-	95.3% (5y)	91.6% (5y)	0.43	-	-	-	-	-	-	39	6	45	54/296	12/97	-	-	-	-	-	-	-	-	-	-
Lemoine et al., 2022 [22]	97 (87.38%)	14 (12.6%)	111	311.4 ± 144.0	287.0 ± 82.7	0.54	(39:58)	(4:10)	(43:68)	22 ± 11	27 ± 8	0.15	443.9 ± 98.6	423.1 ± 70.0	0.39	143.9 ± 122.3 cc/kg	136.1 ± 137.2 cc/kg	0.83	93.8% (1y)	100% (1y)	0.35	14.2 ± 25.6	14.2 ± 10.5	1	7.5 ± 6.6	9.8 ± 9.1	0.27	-	-	-	-	-	-	31.8 ± 36.0	32.8 ± 15.2	0.92	-	-	-	72/99	6/14	-
Chunbao et al., 2010 [23]	9 (41%)	13 (59%)	22	306	204	-	9:0	13:0	22:0	12.3 ± 6.8	19.8 ± 8.5	-	460 ± 75	513 ± 118		260 (150–900) mL	155 (50–650) mL	-	-	-	-	-	-	-	-	-	-	-	-	-	-	-	-	-	-	-	-	-	-	5/9	5/13	-
Choungboonsri et al., 2021 [24]	36 (69.23%)	16 (30.76%)	52	2055±2325	366±99	0.006	-	(16:00)	-	13.1 ± 8.8	20.5 ± 6.7	0.007	605.5 ± 129.6	563.4 ± 74.9	0.235	149.5 ± 248.8 (ml/kg)	117.56 ± 126.93 (ml/kg)	0.635	87.8% (3y)	93.7% (3y)	0.583	9.9 ± 9	6.9 ± 5.9	0.232	-	-	-	3/36	2/16	5/52	5/36	2/16	7/52	45.3 ± 31.4	48.2 ± 38.3	0.772	-	-	-	-	-	-
Karakayali et al., 2008 [25]	14 (70%)	6 (30%)	20	-	-	-	-	-	(18:2)			-	594±126	468±90		2(Unit)	1.4 (unit)	-	-	-	-	-	-	-	0d	0d	0d	4/14	0/6	4/20	2/14	0/6	2/20	-	-	-	-	-	-	2/14	0/6	-
Malhotra et al., 2015 [26]	18 (0.9%)	2 (0.1%)	20	360	210		(18:00)	(2:00)	(20:00)	-	-	-	-	-	-	-	-	-	88.9% (2.5y)	100% (2.5y)		-	-	-	-	-	-	-	-	4	-	-	-	21 d (17–42)	21 d (17–42)	21 d (17–42)	10 (30.3%)	-	-	-	-	-
Safwan et al., 2016 [27]	33 (56.8%)	25 (43.2%)	58	480(150 to 3510)	330 (150 to 1470)	0.011	(33:00)	(25:00)	(58:00)	13 (10 to 28)	17 (4 to 27)	0.043	525.4 ± 125.8	467.5 ± 88.9	0.059	443.9± 169.0	360.4 ±180.6	0.079	93.9% (30day)	100% (30day)	0.5	8 (3 to 45)	6.5 (3 to 10)	0.495	-	-	-	6	4	10	-	-	-	20 (3 to 52)	21 (16 to 45)	0.289	10 (30.3%)	4 (16%)	0.235	-	-	-
Yang et al., 2018 [28]	58 (56.3%)	45 (43.7%)	103	(438.1±716.1)	(225.6±101.3)	0.05	(40:18)	(34:11)	(74:29)	12.8±10.2	19.3±8.8	0.001	384.3±49.6	379.0±48.5	0.89	-	-	-	-	-	-	-	-	-	-	-	-	2	2	4	-	-	-	13.5±1.1	13.6±1.0	0.43	1	2	0.579	0/58	5/40	0.01
Wang et al., 2013 [29]	10 (35.7%)	18 (64.3%)	28	252 ±102	210±120	0.36	(100:00)	(100:00)	(100:00)	11.9±9.2	16±7.4	0.21	534±150	558±252	0.77	197±132.8	208.6±148.5	0.84	80% ( 1y)	77.8% ( 1y)	1	157.7h±37.1	180.4h±44.2	0.18	-	-	-	4	5	9	0/10	0/18	0	29±2	27±4	0.29	-	-	-	6/10	4/18	-

Pooled Analysis

**Figure 2:**
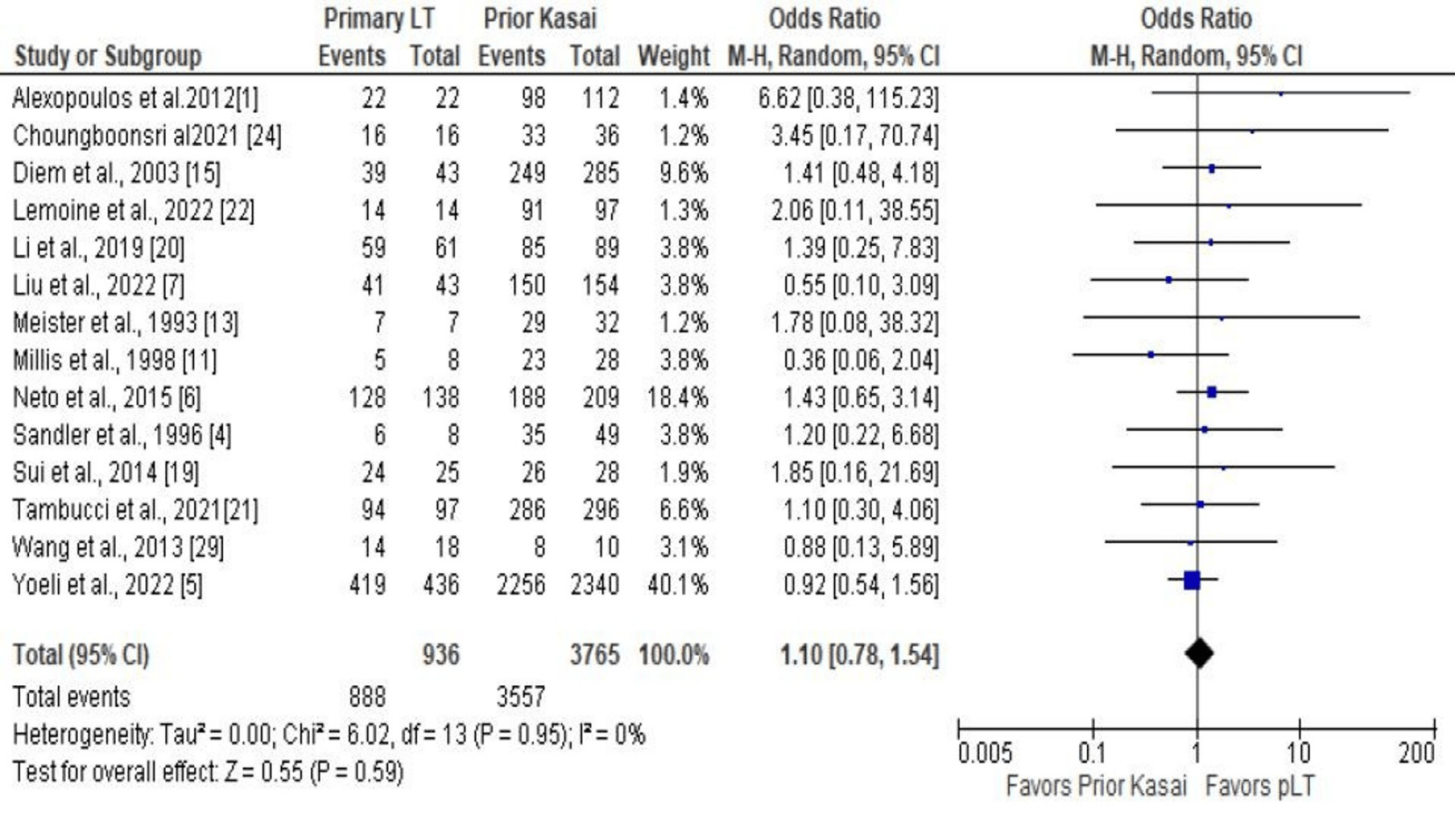
One-year patient survival rate: pLT vs. prior Kasai pLT - primary liver transplant, LT - liver transplant

**Figure 3:**
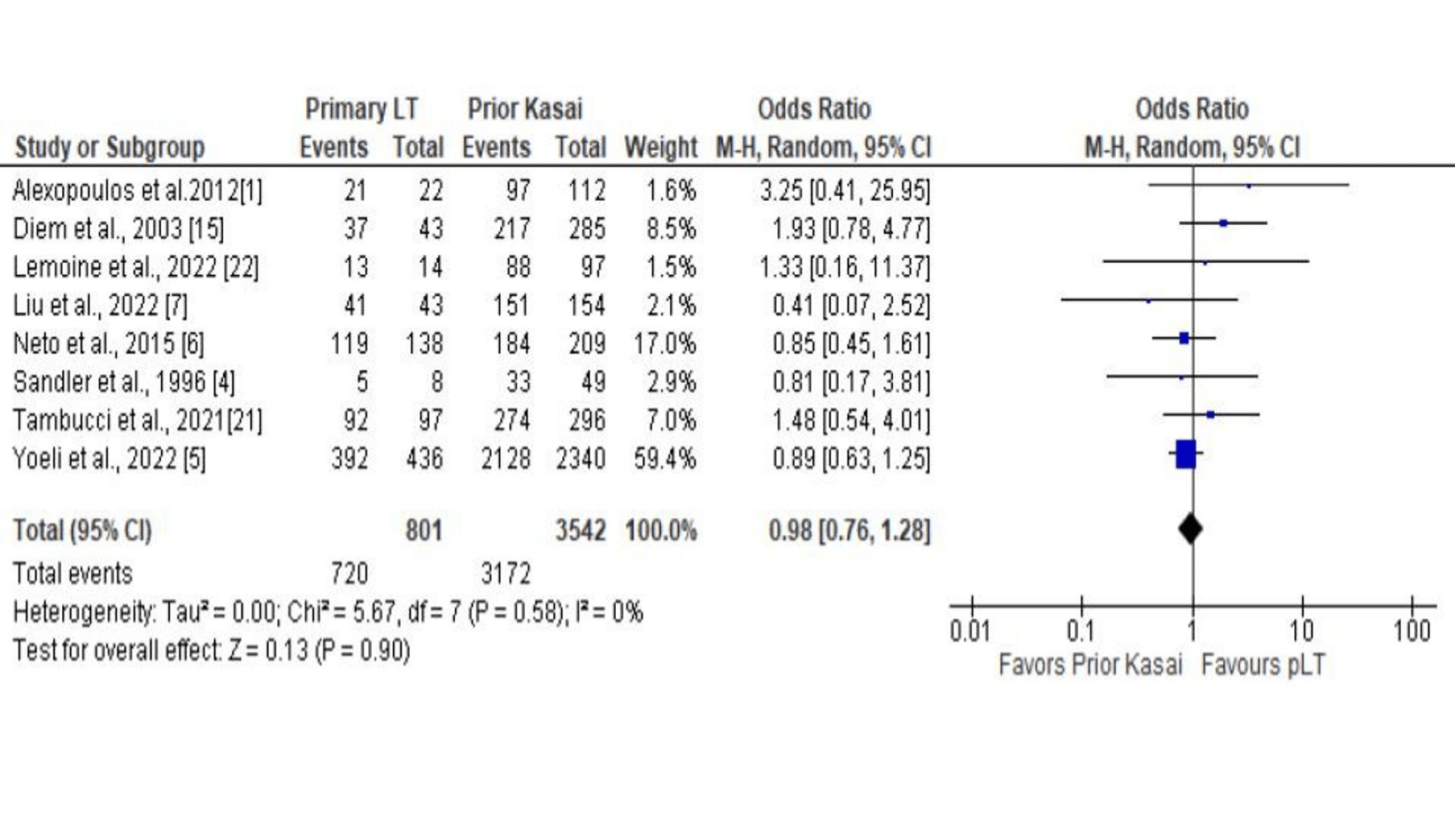
One-year graft survival rate: pLT vs. prior Kasai pLT - primary liver transplant, LT - Liver transplantation

**Figure 4:**
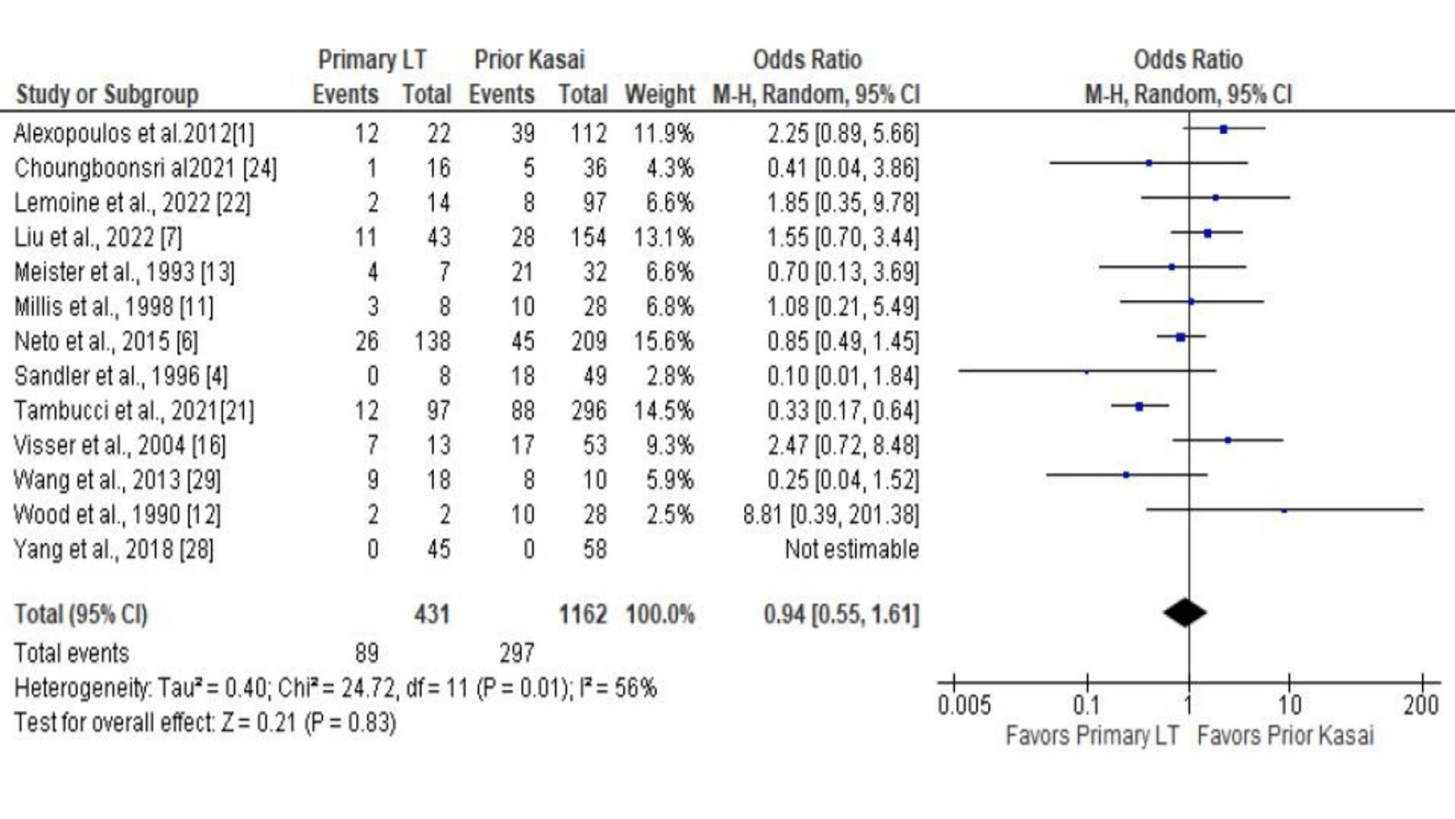
Reoperation rate: pLT vs. prior Kasai pLT - primary liver transplant, LT - Liver transplantation

**Figure 5:**
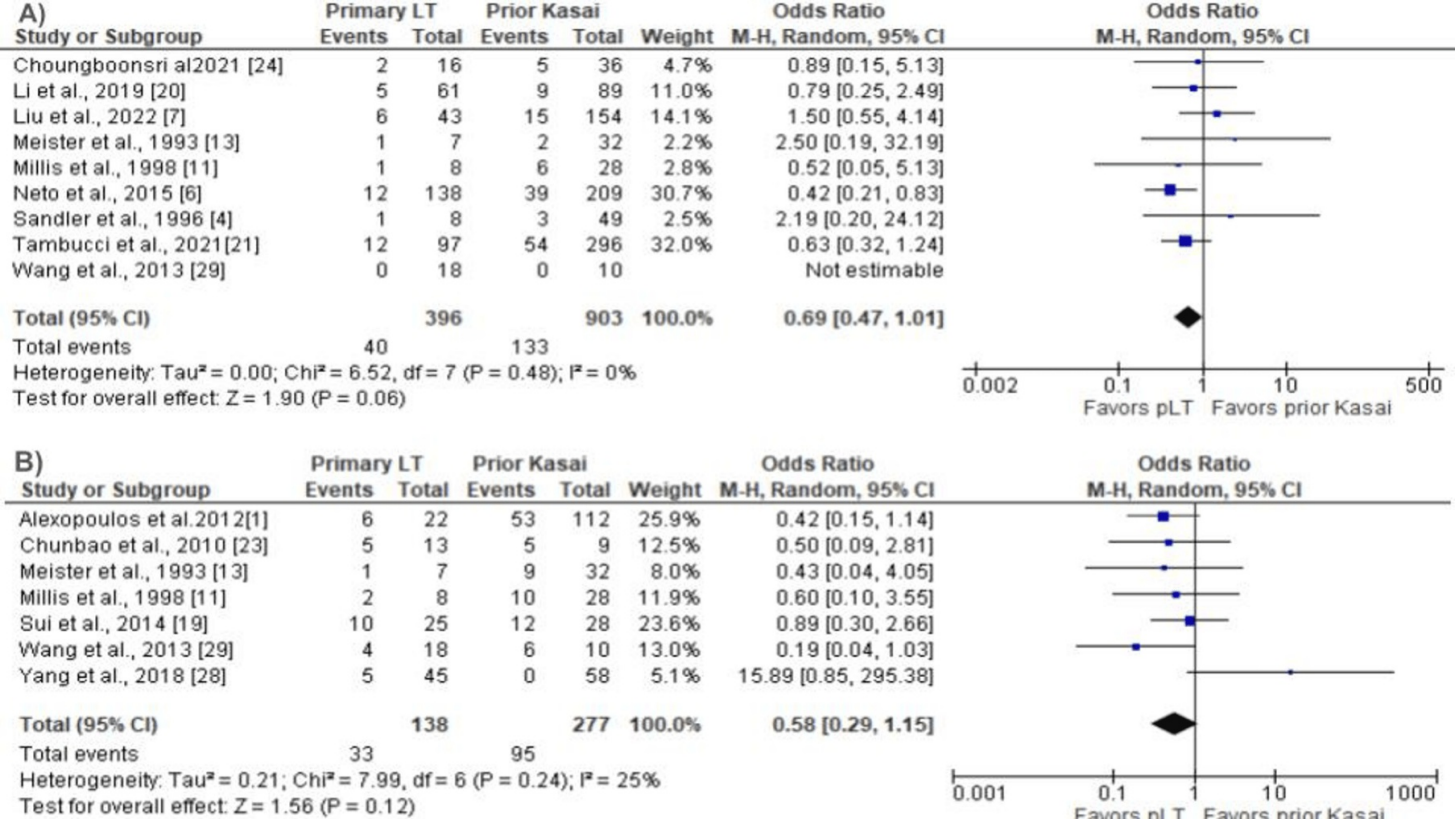
(A) Biliary complication rate: pLT vs. Prior Kasai. (B) Infection rate: pLT vs. prior Kasai

A total of 14 studies reported a one-year patient survival rate, eight reported a one-year graft survival and five-year patient survival rate, five reported five-year graft survival rates, and 13 reported reoperation rates. The meta-analysis did not show a significant difference in the one-year patient survival rate between pLT vs. prior Kasai group (OR 1.10; CI 0.78 - 1.54; P = 0.59; Figure 2) and one-year graft survival rate (OR 0.98; CI 0.76 - 1.28; P = 0.90; Figure 3). The five-year patient and graft survival rates were comparable between the groups (OR 1.07; CI 0.69 - 1.65; P = 0.76 and OR 0.86; CI 0.68 - 1.08; P = 0.20), respectively (Appendix). In addition, there was no statistically significant difference in the reoperation rate (OR 0.94; CI 0.55 - 1.61; P = 0.83; Figure 4). Biliary complications were reduced in the patients with pLT, although the difference was not statistically significant (OR 0.69; CI 0.47 - 1.01; P = 0.06; Figure 5A). Similarly, the infection rate was lower in the pLT group but was not statistically significant (OR 0.58; CI 0.29 - 1.15; P = 0.12; Figure 5B).

Subgroup Analysis

**Figure 6:**
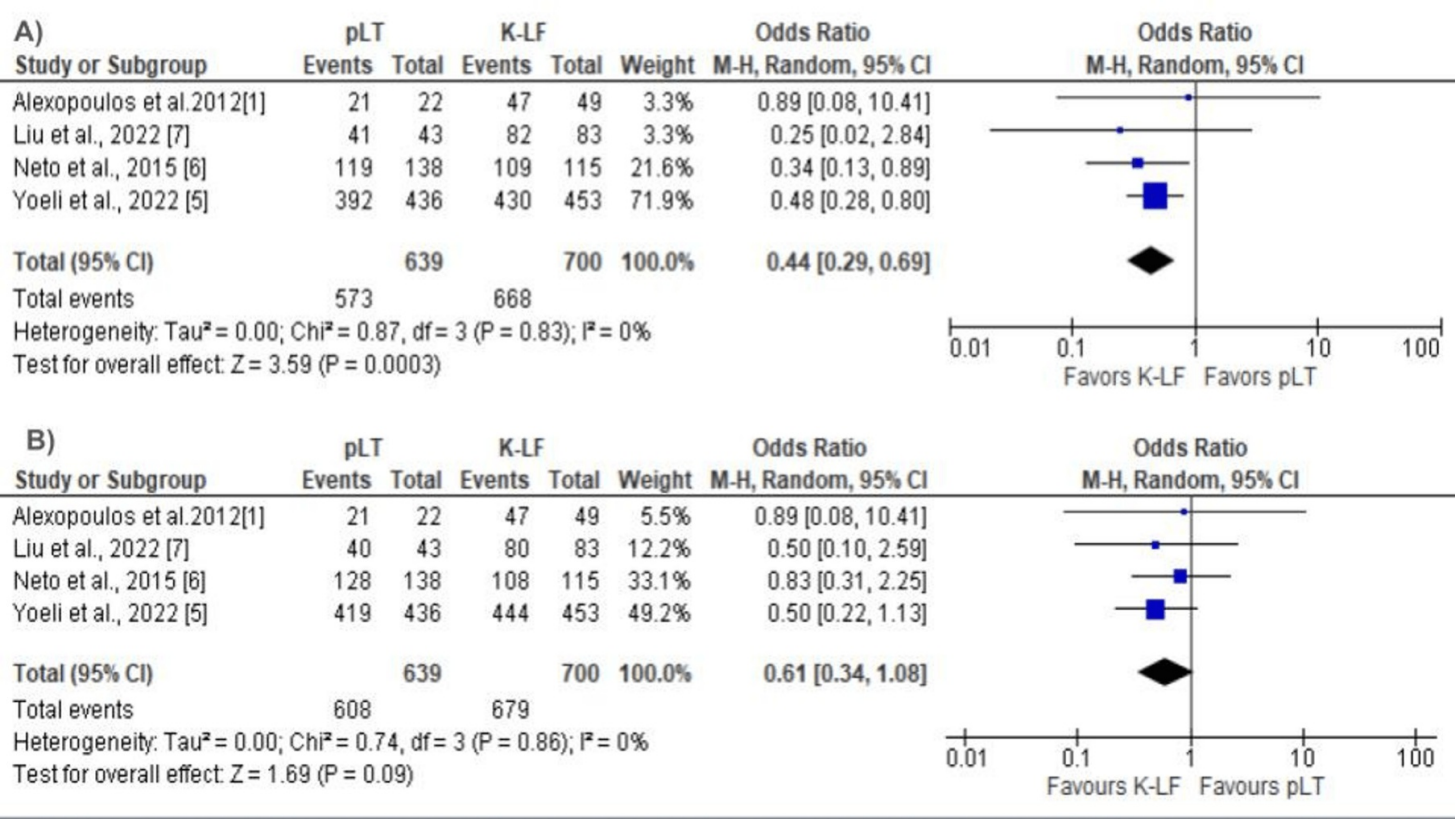
(A) One-year graft survival: pLT vs. K-LF. (B) One-year patient survival: pLT vs. K-LF pLT - primary liver transplant, K-LF - Kasai late failure

Four included studies subcategorized the prior Kasai group into K-EF and K-LF; therefore, we performed a subgroup analysis on patients with pLT and K-LF. The primary LT group had a significantly reduced one-year graft survival compared to the K-LF group (OR 0.44; CI 0.29 - 0.69; P = 0.0003; Figure 6A). One-year patient survival was also decreased in the pLT group, although there was no statistically significant difference (OR 0.61; CI 0.34 - 1.08; P = 0.09; Figure 6B). No statistically significant differences were found for biliary complications and the reoperation rate between patients with pLT and K-LF (OR 1.37; CI 0.31 - 5.96; P = 0.68 and OR 1.55; CI 0.74 - 3.22; P = 0.24), respectively (Appendix). No difference was seen in one-year patient and graft survival when comparing pLT vs. K-EF (OR 1.35; CI 0.53 - 3.42; P = 0.53) and (OR 1.14; CI 0.70 - 1.88; P = 0.59), respectively (Appendix).

Quality Assessment

**Figure 7:**
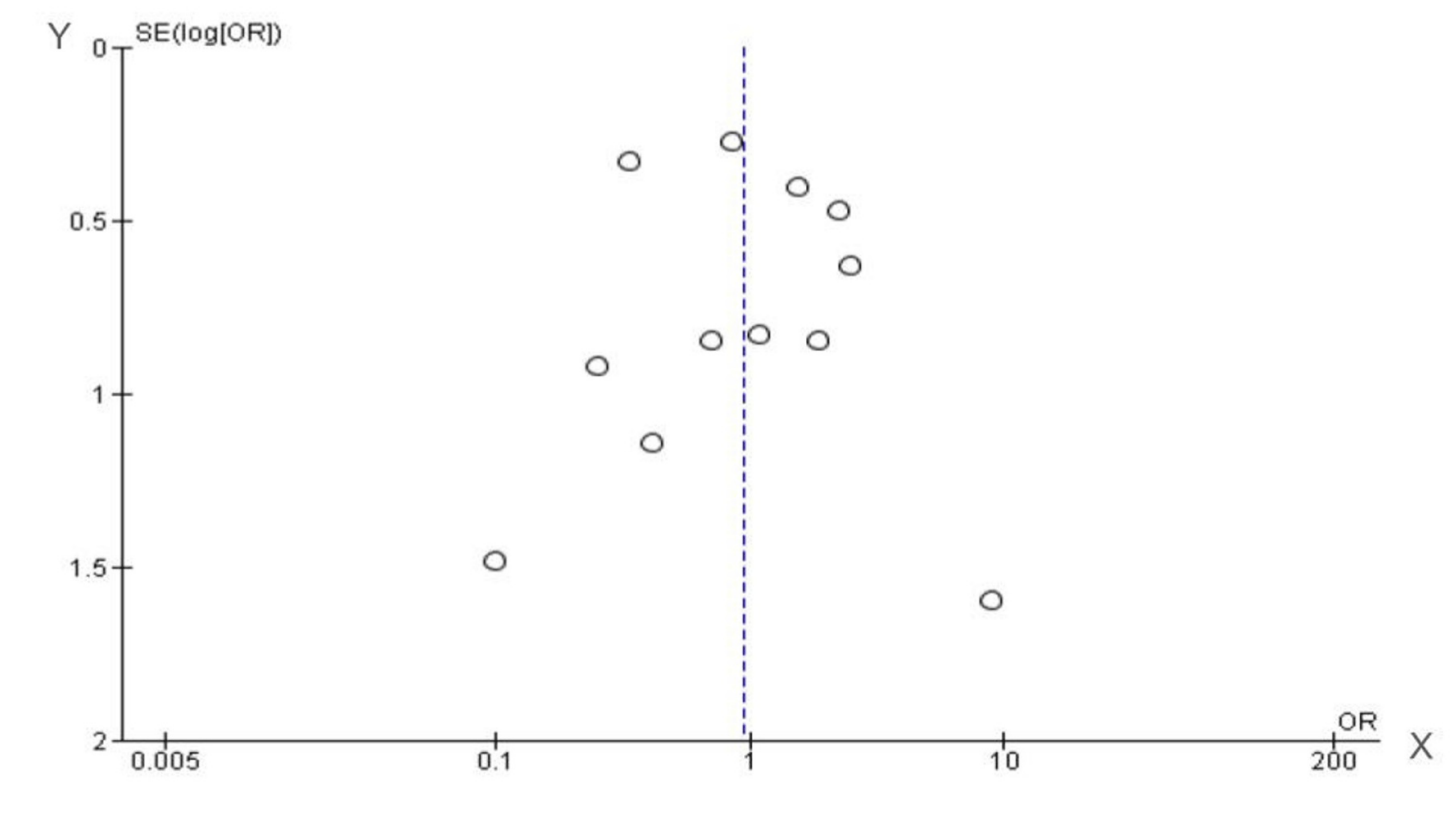
Funnel analysis on reoperation

We included 25 non-randomized studies in this meta-analysis. The appraisal of each individual study is reported in Table 2 for non-RCTs. In general, most of the studies were classified as having a moderate risk of bias and two as a critical risk. There was no evidence of publication bias by funnel plot analysis (Figure 7).

**Table 2:** Risk of bias summary for non-randomized studies (ROBINS-I)

Study	Bias due to confounding	Bias in selection of participants	Bias in classification of interventions	Bias due to deviations from intended interventions	Bias due to missing data	Bias in measurement of outcomes	Bias in selection of the reported result	Overall risk of bias judgement
Yoeli et al., 2022 [5]	Moderate	Low	Low	Low	Low	Low	Low	Moderate
Yang et al., 2018 [28]	Moderate	Low	Low	Low	Low	Low	Low	Moderate
Wood et al., 1990 [12]	Moderate	Low	Low	Low	Moderate	Low	Low	
Wang et al., 2013 [29]	Moderate	Low	Low	Low	Low	Low	Low	Moderate
Visser et al., 2004 [16]	Moderate	Low	Low	Low	Moderate	Low	Low	Moderate
Tiao et al., 2008 [18]	Moderate	Low	Low	Low	Low	Low	Low	Moderate
Tambucci et al., 2021 [21]	Moderate	Low	Low	Low	Low	Low	Low	Moderate
Sandler et al., 1996 [4]	Moderate	Low	Low	Low	Low	Low	Low	Moderate
Safwan et al., 2016 [27]	Moderate	Low	Low	Low	Low	Low	Low	Moderate
Neto et al., 2015 [6]	Moderate	Low	Low	Low	Low	Low	Low	Moderate
Millis et al., 1998 [11]	Moderate	Low	Low	Low	Low	Low	Low	Moderate
Meister et al., 1993 [13]	Moderate	Moderate	Low	Low	Low	Low	Low	Moderate
Malhotra et al., 2015 [26]	Moderate	Low	Low	Low	Low	Low	Low	Moderate
Liu et al., 2022 [7]	Moderate	Low	Low	Low	Low	Low	Low	Moderate
Li et al., 2019 [20]	Moderate	Low	Low	Low	Low	Low	Low	Moderate
Lemoine et al., 2022 [22]	Moderate	Low	Low	Low	Low	Low	Low	Moderate
LeeVan et al., 2018 [2]	Moderate	Low	Low	Low	Serious	Moderate	Low	Serious
Karakayali et al., 2008 [25]	Moderate	Low	Low	Low	Low	Low	Low	Moderate
Diem et al., 2003 [15]	Moderate	Low	Low	Low	Low	Low	Low	Moderate
Cowles et al., 2008 [17]	Moderate	Low	Low	Low	Low	Low	Low	Moderate
Choungboonsri et al., 2021 [24]	Moderate	Low	Low	Low	Low	Low	Low	Moderate
Chardot et al., 2001 [14]	Moderate	Low	Low	Low	Serious	Low	Low	Serious
Alexopoulos et al., 2012 [1]	Moderate	Low	Low	Low	Low	Low	Low	Moderate

Discussion

This systematic review and meta-analysis of 25 studies and 6,408 BA patients compared the outcomes of LT in infants with prior Kasai portoenterostomy versus primary LT. The main findings were as follows: (1) comparable outcomes were seen in one-year patient and graft survival between pLT vs. prior Kasai; (2) no difference was seen in the patient and graft five-year survival and reoperation rate; (3) subgroup analysis demonstrated a significant reduction in one-year graft survival in the pLT group compared to K-LF; there was a reduced although a not significant difference in one-year patient survival in pLT compared to K-LT; (4) comparable outcomes in reoperation and biliary complications were seen between the two groups; and (5) there were no differences in the outcomes after pLT or K-EF.

The Kasai procedure is the preferred initial treatment for infants with BA. Primary liver transplants for BA are usually reserved for advanced diseases at the time of diagnosis [30]. Many patients who undergo the Kasai procedure will eventually fail and require a salvage LT (sLT). The high failure rate of the Kasai procedure and advances in the pediatric liver transplant field with an excellent outcome led to the question of whether the Kasai procedure should remain the initial treatment for infants with BA. Studies have investigated the influence of prior Kasai procedure on the outcomes of LT. LeeVan et al., in their retrospective analysis of 313 pLT patients and 147 sLT, reported a higher risk of mortality in patients who had prior Kasai compared to pLT (HR 0.43; P = 0.03). Similarly, higher post-transplant surgical complications were reported in a prior Kasai group by Tambucci et al. On the contrary, several other studies reported no significant difference in the patient and graft survival rate [5,21,22]. No significant statistical difference was seen in the patient and graft survival rates between pLT and prior Kasai group in this meta-analysis (P = 0.59 and P = 0.90), respectively. In addition, there was no difference in the reoperation rate.

Yang et al. investigated the impact of the Kasai procedure and nutritional status. They reported a significantly lower incidence of severe pulmonary infection in the prior Kasai group than pLT (P = 0.01). Similarly, Liu et al. reported a higher incidence of cytomegalovirus infection in patients in the pLT group. Conversely, the prior Kasai group was reported to have an increased infection complication rate and higher mortality risk than the pLT group by LeeVan et al. We report a reduced infection rate in the pLT group, although this was not statistically significant between the groups (OR 0.58; CI 0.25 - 1.15; P = 0.12).

Previous meta-analyses did not include biliary complications in the analysis. However, it is an important complication in pediatric LT, with a rate of approximately 15% according to Registry data from pediatric-focused liver transplant programs in North America [31]. There is heterogeneity in the reports of biliary complications; some studies reported no difference in the incidence of biliary complications between groups [7,24], whereas others reported a lower incidence of biliary complications in the pLT group [6]. Cumulative data demonstrates non-statistically significant differences in the incidence of biliary complications between pLT and the prior Kasai group (OR 0.69; 0.47 - 1.01; P = 0.06).

Two distinct populations can be seen in the patients who underwent prior Kasai group. However, most studies have not distinguished between these patients when performing analysis, which might have contributed to the heterogeneity of the outcomes reported across the studies [1-5,11-28]. Patients with K-EF tend to display similar characteristics to pLT at the time of transplantation, such as lower body weight and higher PELD score, in contrast to the K-LF group [1;5-7]. Additionally, lower body weight is known to be predictive of reduced patient and graft survival rates after LT [32]. A subanalysis of pLT vs. K-LF reported a 56% decrease in the odds of one-year graft survival in the pLT group (P = 0.0003) and a 39% decrease in the odds of patient survival after pLT. However, comparable outcomes were seen when comparing pLT to K-EF.

This study had some limitations. First, our meta-analysis included retrospective studies that were susceptible to bias in all retrospective studies. Second, most of the included studies had a moderate risk of bias. Third, the population number in this study may still be unpowered to detect differences in the major outcomes between pLT and prior Kasai.

## Conclusions

Our study investigated posttransplant outcomes in BA patients with prior Kasai procedure and pLT. In addition, we subcategorized prior K-EF and K-LF and compared their outcomes to the pLT group. This study found no difference in one- and five-year patient and graft survival outcomes between pLT vs. prior Kasai group. However, we did find decreased odds of a one-year graft survival rate in pLT compared to the K-LF group. According to our results, K-EF and pLT presented comparable survival outcomes, and K-LF presented a superior graft survival rate to the pLT groups. These results support the findings that patients with prior Kasai are clinically distinctive and may have different transplant outcomes depending on the time of their initial Kasai procedure failure.

## Figures and Tables

**Figure 8:**
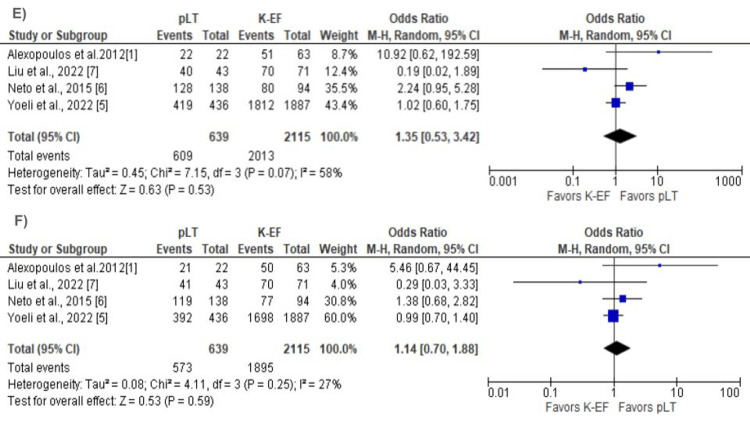
Supplemental Appendix (E) One-year patient survival rate: pLT vs. Kasai late failure (K-EF). (F) One-year graft survival rate: pLT vs. Kasai late failure (K-EF).

**Figure 9:**
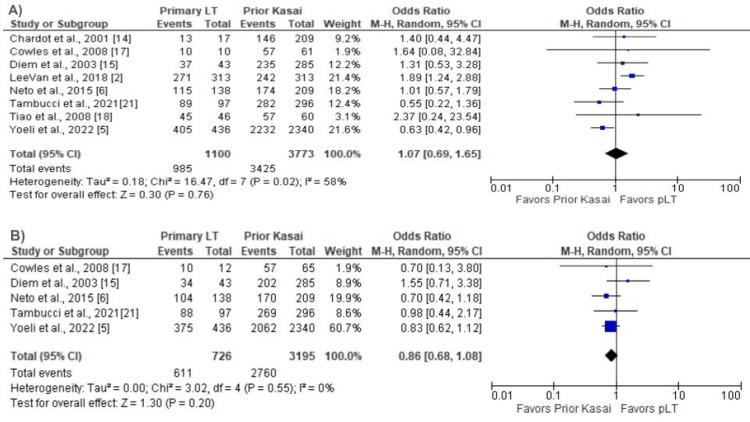
Supplemental Appendix (A) Five-year patient survival rate: pLT vs. prior Kasai. (B) Five-year graft survival rate: pLT vs. prior Kasai.

**Figure 10:**
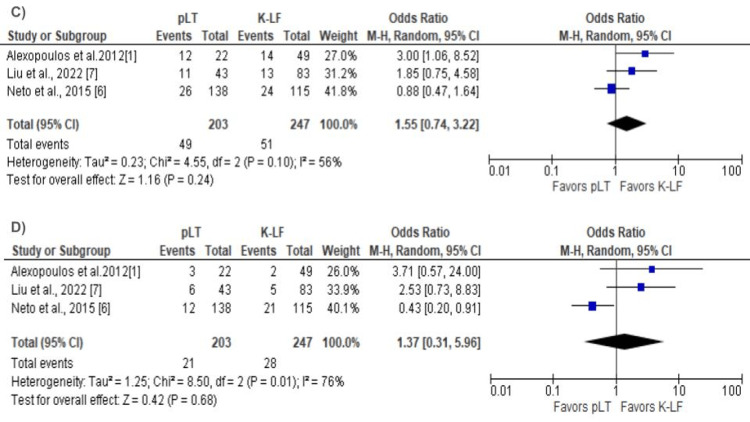
Supplemental Appendix (C) Reoperation rate: pLT vs. Kasai late failure (K-LF). (D) Biliary complication rate: pLT vs. Kasai late failure (K-LF).
