# Correction: Are the Post-transplant Outcomes of Kasai's Early Failure and Late Failure Comparable to the Primary Liver Transplantation?

**DOI:** 10.7759/cureus.c355

**Published:** 2025-10-13

**Authors:** Valberto Sanha, Tales A Franzini, Waldemir F Junior, Antonio N Kalil

**Affiliations:** 1 General Surgery, Federal University of Health Science of Porto Alegre, Porto Alegre, BRA; 2 Medicine, Federal University of Health Science of Porto Alegre, Porto Alegre, BRA; 3 Surgery, Federal University of Health Science of Porto Alegre, Porto Alegre, BRA; 4 General Surgery and Hepato-Pancreato-Biliary (HPB) & Transplant Surgery, Federal University of Health Science of Porto Alegre, Porto Alegre, BRA

This article has been corrected to fix the following typo in the PRISMA diagram and preceding paragraph: 'Records identified from citation searching' has been corrected from 3 to 4.

